# Supplementary material for: The intrinsically disordered regions of organellophagy receptors are interchangeable and control organelle fragmentation, ER-phagy and mitophagy flux
Source: Nat Cell Biol. 2025 Aug 4;27(9):1431–47. doi: 10.1038/s41556-025-01728-4 (PMC12431859; doi:10.1038/s41556-025-01728-4)
Supplement: Supplementary file 1 — Reporting Summary [file 41556_2025_1728_MOESM1_ESM.pdf]

nature portfolio

Corresponding author(s): Maurizio Molinari

Last updated by author(s): May 6, 2025

Reporting Summary

Nature Portfolio wishes to improve the reproducibility of the work that we publish. This form provides structure for consistency and transparency in reporting. For further information on Nature Portfolio policies, see our Editorial Policies and the Editorial Policy Checklist.

Statistics

For all statistical analyses, confirm that the following items are present in the figure legend, table legend, main text, or Methods section.

n/a	Confirmed	
☐	☒	The exact sample size (*n*) for each experimental group/condition, given as a discrete number and unit of measurement
☐	☒	A statement on whether measurements were taken from distinct samples or whether the same sample was measured repeatedly
☐	☒	The statistical test(s) used AND whether they are one- or two-sided<br>*Only common tests should be described solely by name; describe more complex techniques in the Methods section.*
☒	☐	A description of all covariates tested
☐	☒	A description of any assumptions or corrections, such as tests of normality and adjustment for multiple comparisons
☐	☒	A full description of the statistical parameters including central tendency (e.g. means) or other basic estimates (e.g. regression coefficient) AND variation (e.g. standard deviation) or associated estimates of uncertainty (e.g. confidence intervals)
☐	☒	For null hypothesis testing, the test statistic (e.g. *F*, *t*, *r*) with confidence intervals, effect sizes, degrees of freedom and *P* value noted<br>*Give P values as exact values whenever suitable.*
☒	☐	For Bayesian analysis, information on the choice of priors and Markov chain Monte Carlo settings
☒	☐	For hierarchical and complex designs, identification of the appropriate level for tests and full reporting of outcomes
☒	☐	Estimates of effect sizes (e.g. Cohen's *d*, Pearson's *r*), indicating how they were calculated

Our web collection on statistics for biologists contains articles on many of the points above.

Software and code

Policy information about availability of computer code

Data collection	Immunoflurescence data: Leica TCS SP5 confocal system and Leica Stellaris SP8 microscope with Leica LAS X 4.5.0.025531<br>Western blotting data: FusionFX7 VILBER (Witec) with Fusion FX7 Edge 18.12 software<br>Fluorescent gels: Amersham Typhoon scanner (Cytiva)<br>Immunogold electron microscopy: Transmission Electron Microscope Talos L120C (FEI, Thermo Fisher Scientific) with a Ceta CCD camera (FEI, Thermo Fisher Scientific), Velox 3.6.0 software (FEI, Thermo Fisher Scientific)<br>RT-Electron Tomography : Leica Stellaris SP8 Leica LAS X 4.5.0.025531 software, Transmission Electron Microscope Talos L120C (FEI, Thermo Fisher Scientific) with Tomography 5 software (FEI, Thermo Fisher Scientific).<br>LC/MS: Vanquish Neo nanoHPLC system interfaced via a nanospray Flex source to a high resolution Orbitrap Exploris 480 mass spectrometer (Thermo Fisher Scientific) and Xcalibur software (Tune 2.9, Thermo Fisher Scientific)
Data analysis	MaxQuant 2.1.4.0<br>ImageJ 2.16.0/1.54p for windows64<br>LysoQuant plugin for ImageJ (an unbiased and automated deep learning tool for fluorescent image quantification, which is freely available (https://www.irb.usi.ch/lysoquant/))<br>IMOD 4.11.24<br>Microscopy Image Browser (MIB) 2.84<br>Graphing and statistic analysis were performed using GraphPad PRISM 10 (10.1.2 for Windows)<br>Photoshop 26.2.0

Matlab R2023b Update 5 23.2.0.24591.
Data analysis for proteomics data was performed in Perseus v2.0.5

For manuscripts utilizing custom algorithms or software that are central to the research but not yet described in published literature, software must be made available to editors and reviewers. We strongly encourage code deposition in a community repository (e.g. GitHub). See the Nature Portfolio guidelines for submitting code & software for further information.

Data

Policy information about availability of data

All manuscripts must include a data availability statement. This statement should provide the following information, where applicable:

- Accession codes, unique identifiers, or web links for publicly available datasets
- A description of any restrictions on data availability
- For clinical datasets or third party data, please ensure that the statement adheres to our policy

The MS proteomics data supporting the data in Ext. Fig. 1f and Ext. Fig. 1g can be accessed from the ProteomeXchange Consortium via the PRIDE partner repository under accession code PXD060519. Numerical data used to create graphs is included as Source data tables. Uncropped blots and gels are included in Image source data. No restrictions on data availability apply.

Research involving human participants, their data, or biological material

Policy information about studies with human participants or human data. See also policy information about sex, gender (identity/presentation), and sexual orientation and race, ethnicity and racism.

Reporting on sex and gender	N/A
Reporting on race, ethnicity, or other socially relevant groupings	N/A
Population characteristics	N/A
Recruitment	N/A
Ethics oversight	N/A

Note that full information on the approval of the study protocol must also be provided in the manuscript.

Field-specific reporting

Please select the one below that is the best fit for your research. If you are not sure, read the appropriate sections before making your selection.

☒ Life sciences ☐ Behavioural & social sciences ☐ Ecological, evolutionary & environmental sciences

For a reference copy of the document with all sections, see nature.com/documents/nr-reporting-summary-flat.pdf

Life sciences study design

All studies must disclose on these points even when the disclosure is negative.

Sample size	No statistical methods were used to pre-determine sample sizes but our sample sizes are similar to those reported in previous publications (DOIs: 10.15252/embj.2020107240; 10.15252/embj.201899259; 10.1091/mbc.E20-04-0269; 10.1038/s41467-023-39172-3; 10.1091/mbc.E21-10-0526).
Data exclusions	No data were excluded from the analyses.
Replication	The number of cells analyzed and the number of independent experiments is specified for each experiment in the figure legends.
Randomization	No randomization was performed as this is not common for western blot or microscopy analysis. Covariates were controlled with the corresponding negative controls.
Blinding	Unbiased imaging data collection/analyses were performed by at least two different scientists, one of which was blind to the identity of the sample.<br>Morphological analyses of mitochondrial size were performed by the scientists blinded to the identity of the sample. The fluorescent image quantifications of ER and mitochondria delivery to endolysosomes were performed using LysoQuant, an unbiased and automated deep learning tool. Acquisition of MS data was done by scientist blinded to the nature of the sample and the scope of the experiment.<br>For WB and IP analyses, conclusions were drawn based on qualitative presence/absence of band signal, therefore, blinding is not relevant.<br>For electron microscopy, acquisition of samples was performed by a scientist blinded to the nature of the sample.

Reporting for specific materials, systems and methods

We require information from authors about some types of materials, experimental systems and methods used in many studies. Here, indicate whether each material, system or method listed is relevant to your study. If you are not sure if a list item applies to your research, read the appropriate section before selecting a response.

Materials & experimental systems

n/a	Involved in the study
☐	☒ Antibodies
☐	☒ Eukaryotic cell lines
☒	☐ Palaeontology and archaeology
☒	☐ Animals and other organisms
☒	☐ Clinical data
☒	☐ Dual use research of concern
☒	☐ Plants

Methods

n/a	Involved in the study
☒	☐ ChIP-seq
☒	☐ Flow cytometry
☒	☐ MRI-based neuroimaging

Antibodies

Antibodies used

Rat anti-LAMP1 DSHB 1D4B 1:50 (CLSM)
Rabbit anti-TOMM20 Abcam ab186734 1:100 (CLSM)
Rabbit anti-LC3B Sigma L7543 1:1000 (IB)
Mouse anti-LAMP1 DSHB H4A3 1:100 (CLSM)
Rabbit Anti-ATG7 Sigma A2856 1:600 (IB)
Rabbit Anti-DRP1 Abcam ab184247 1:1000 (IB)
Rabbit anti-GFP Abcam ab290 1:50 (IEM) 1:1500 (IB)
Mouse anti-GAPDH Millipore MAB374 clone C 1:30000 (IB)
Protein A HRP-conjugated Invitrogen 101023 1:20000 (IB)
Goat anti-rabbit AlexaFluor488-conjugated Thermo Fisher Scientific A-21206 1:300 (CLSM)
Goat anti-rabbit AlexaFluor568-conjugated Thermo Fisher Scientific A-11036 1:300 (CLSM)
Goat anti-rat AlexaFluor647-conjugated Thermo Fisher Scientific A-21247 1:300 (CLSM)
Goat anti-rabbit AlexaFluor405-conjugated Thermo Fisher Scientific A-31556 1:150 (CLSM)
Goat anti-rabbit gold-labelled Nanoprobes 2004 1:100 (IEM)
Rabbit anti-CNX Kind gift from A. Helenius Not applicable 1:100 (CLSM)

Validation

Anti-ATG7 and anti-DRP1 antibodies were validated with western blots using KO cell lines. Anti-LC3B antibody in WB by observing 2 characteristic bands corresponding to the lipidated and non-lipidated form at the corresponding MWs. Primary Anti-GFP antibody was validated in overexpression experiments using sfGFP-tagged proteins. Anti-TOMM20 antibody was validated by CLSM by observing the signal colocalizing with mitochondria (MitoTracker). Anti-CNX, anti-TOMM20 and anti-LAMP1 antibodies were validated by CLSM by colocalization with the Endoplamsic Reticulum, mitochondria (MitoTracker) and endolysosomes, respectively. Anti-GAPDH antibody was validated by WB with the only band visible corresponding to the expected MW.

Eukaryotic cell lines

Policy information about cell lines and Sex and Gender in Research

Cell line source(s)

MEF WT and Atg7KO are kind gift from M Kumatsu, HEK293 were purchased from ATCC (CRL-1573). WT and DRP1KO MEFs are kind gift from S. Manley and M. Ryan

Authentication

ATG7KO were checked for absence of ATG7 and for lack of LC3 lipidation (e.g., Komatsu, M. et al. JCB 2005; Fumagalli, F. et al. Nature Cell Biol 2016; Fasana, E. et al EMBO Rep. 2024). DRP1KO MEFs were checked for abscence of DRP1 and enlarged mitochondria. WT MEF cells resulted positive for DRP1 and ATG7, displayed classical fibroblast morphology and were positive for LAMP1 staining with mouse-specific LAMP1 antibody. HEK293 were obtained from ATCC, and their identity was confirmed by morphology.

Mycoplasma contamination

The cell lines were tested regularly to be negative for mycoplasma contamination.

Commonly misidentified lines
(See ICLAC register)

Commonly misidentified lines were NOT used in this study.

Plants

Seed stocks	N/A
Novel plant genotypes	N/A
Authentication	N/A
