# Supplementary material for: The intrinsically disordered regions of organellophagy receptors are interchangeable and control organelle fragmentation, ER-phagy and mitophagy flux
Source: Nat Cell Biol. 2025 Aug 4;27(9):1431–47. doi: 10.1038/s41556-025-01728-4 (PMC12431859; doi:10.1038/s41556-025-01728-4)
Supplement: Supplementary file 8 — Details of antibodies and fluorescent ligands used. IB, immunoblotting. [file 41556_2025_1728_MOESM8_ESM.pdf]

Details of antibodies and fluorescent ligands used. IB: Immunoblotting; CLSM: Confocal Laser Scanning Microscopy; IEM: Immunoelectron Microscopy.

Antibody	Manufacturer	Catalog nr	Dilution or Concentration
Rat anti-LAMP1	DSHB	1D4B	1:50 (CLSM)
Rabbit anti-TOMM20	Abcam	ab186734	1:100 (CLSM)
Rabbit anti-LC3	Sigma	L7543	1:1000 (IB)
Mouse anti-LAMP1	DSHB	H4A3	1:100 (CLSM)
Rabbit anti-CNX	Kind gift from A. Helenius	Not applicable	1:100 (CLSM)
Rabbit Anti-ATG7	Sigma	A2856	1:600 (IB)
Rabbit Anti-DRP1	Abcam	ab184247	1:1000 (IB)
Rabbit anti-GFP	Abcam	ab290	1:50 (IEM) 1:1500 (IB)
Mouse anti-GAPDH	Millipore	MAB374 clone 6C5	1:30000 (IB)
Protein A HRP-conjugated	Invitrogen	101023	1:20000 (IB)
Goat anti-rabbit AlexaFluor488-conjugated	Thermo Fisher Scientific	A-21206	1:300 (CLSM)
Goat anti-rabbit AlexaFluor568-conjugated	Thermo Fisher Scientific	A-11036	1:300 (CLSM)
Goat anti-rat AlexaFluor647-conjugated	Thermo Fisher Scientific	A-21247	1:300 (CLSM)
Goat anti-rabbit AlexaFluor405-conjugated	Thermo Fisher Scientific	A-31556	1:150 (CLSM)
Goat anti-rabbit gold-labelled	Nanoprobes	2004	1:100 (IEM)
