# Supplementary figures and images for: The intrinsically disordered regions of organellophagy receptors are interchangeable and control organelle fragmentation, ER-phagy and mitophagy flux
Source: Nat Cell Biol. 2025 Aug 4;27(9):1431–47. doi: 10.1038/s41556-025-01728-4 (PMC12431859; doi:10.1038/s41556-025-01728-4)

Figure 2

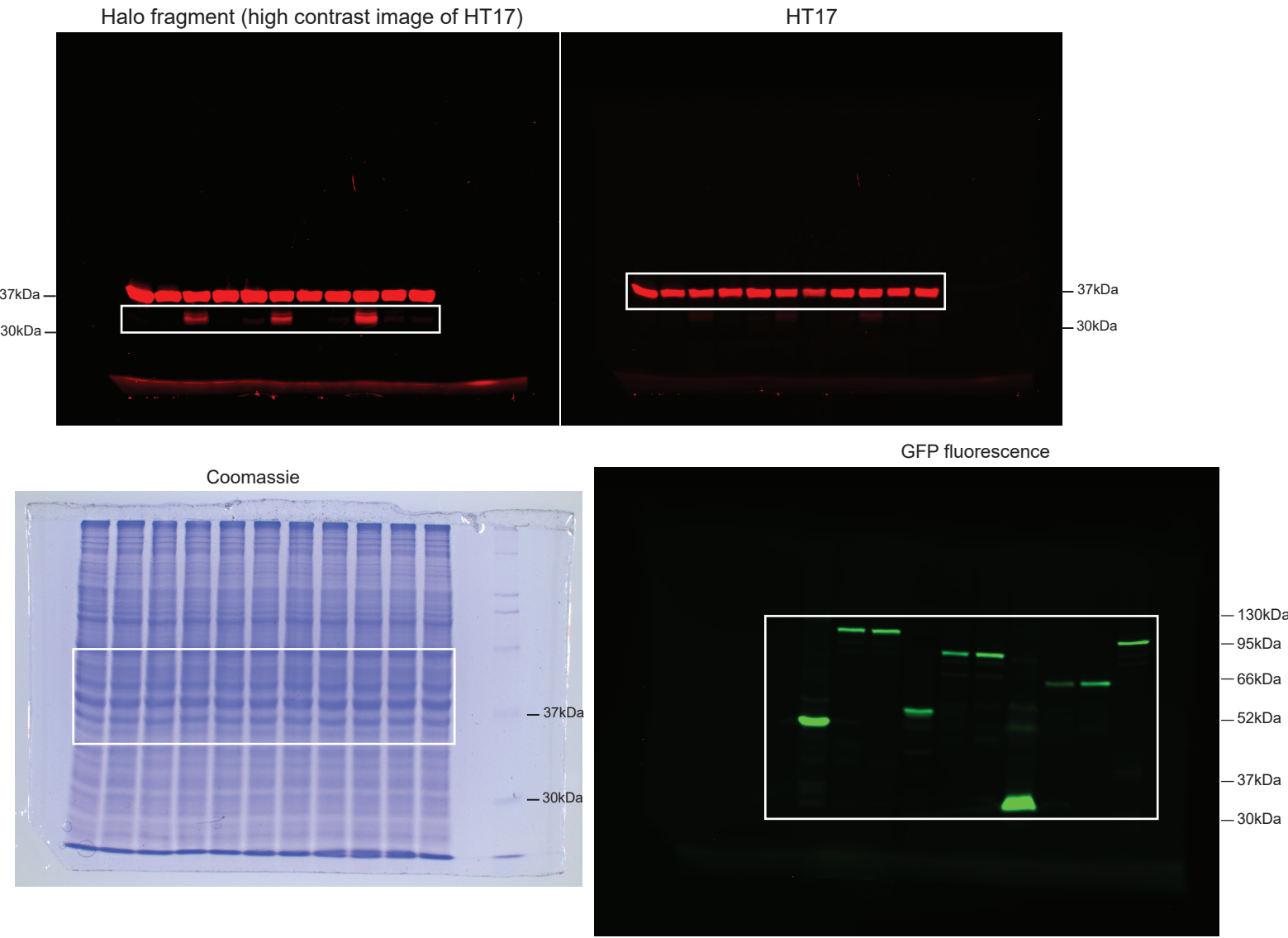

Supplement: Supplementary file 10 — Unprocessed western blots and/or gels. [file 41556_2025_1728_MOESM10_ESM.pdf]

Figure 3

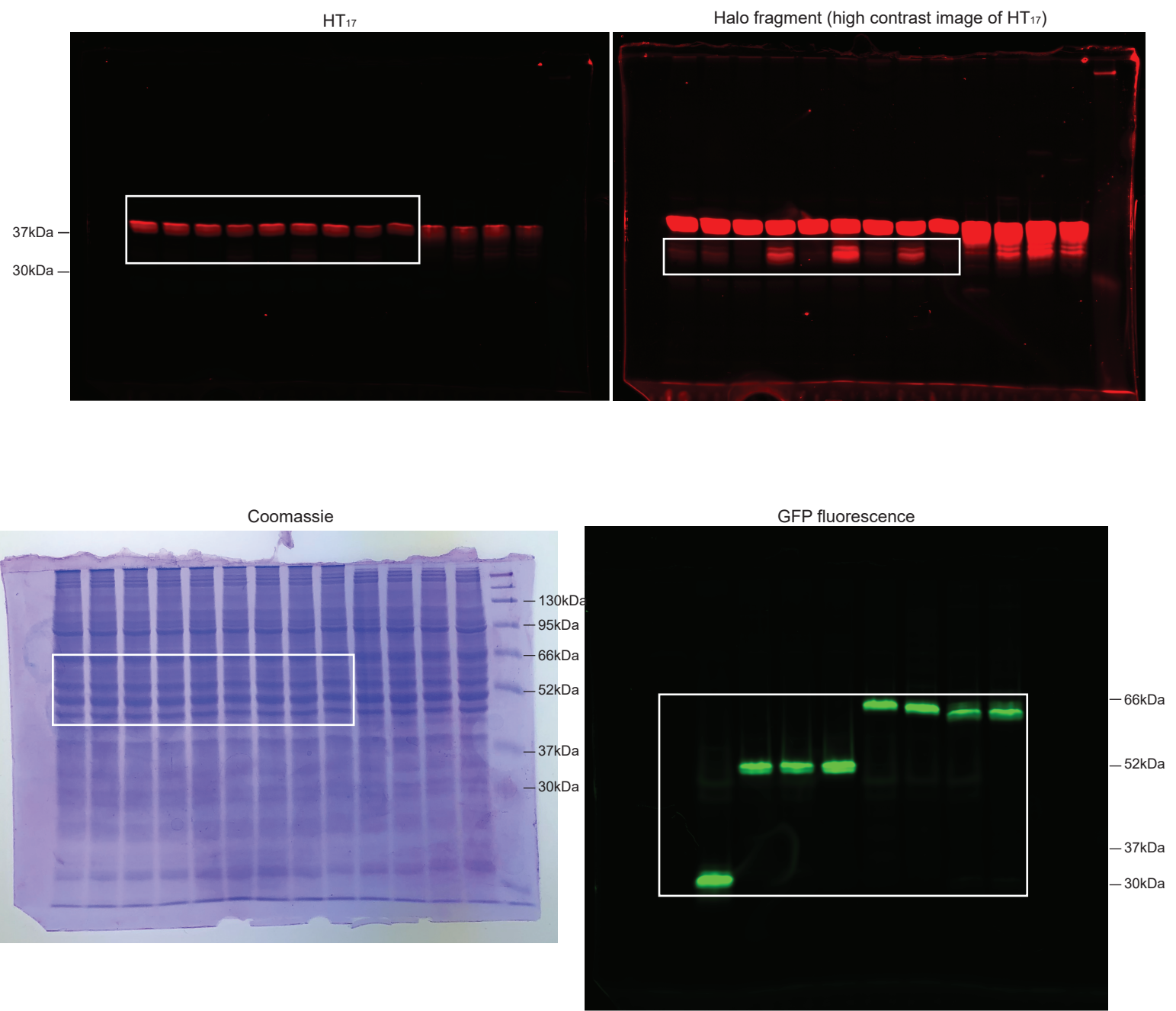

Supplement: Supplementary file 11 — Unprocessed western blots and/or gels. [file 41556_2025_1728_MOESM11_ESM.pdf]

Figure 4

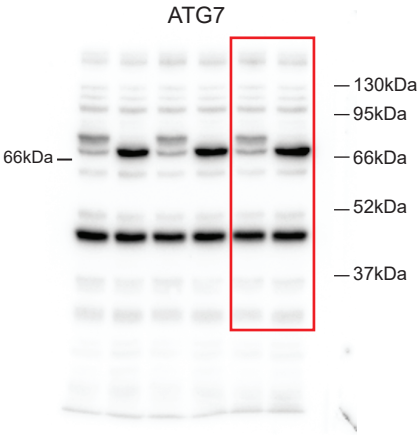

Supplement: Supplementary file 12 — Unprocessed western blots and/or gels. [file 41556_2025_1728_MOESM12_ESM.pdf]

Figure 5

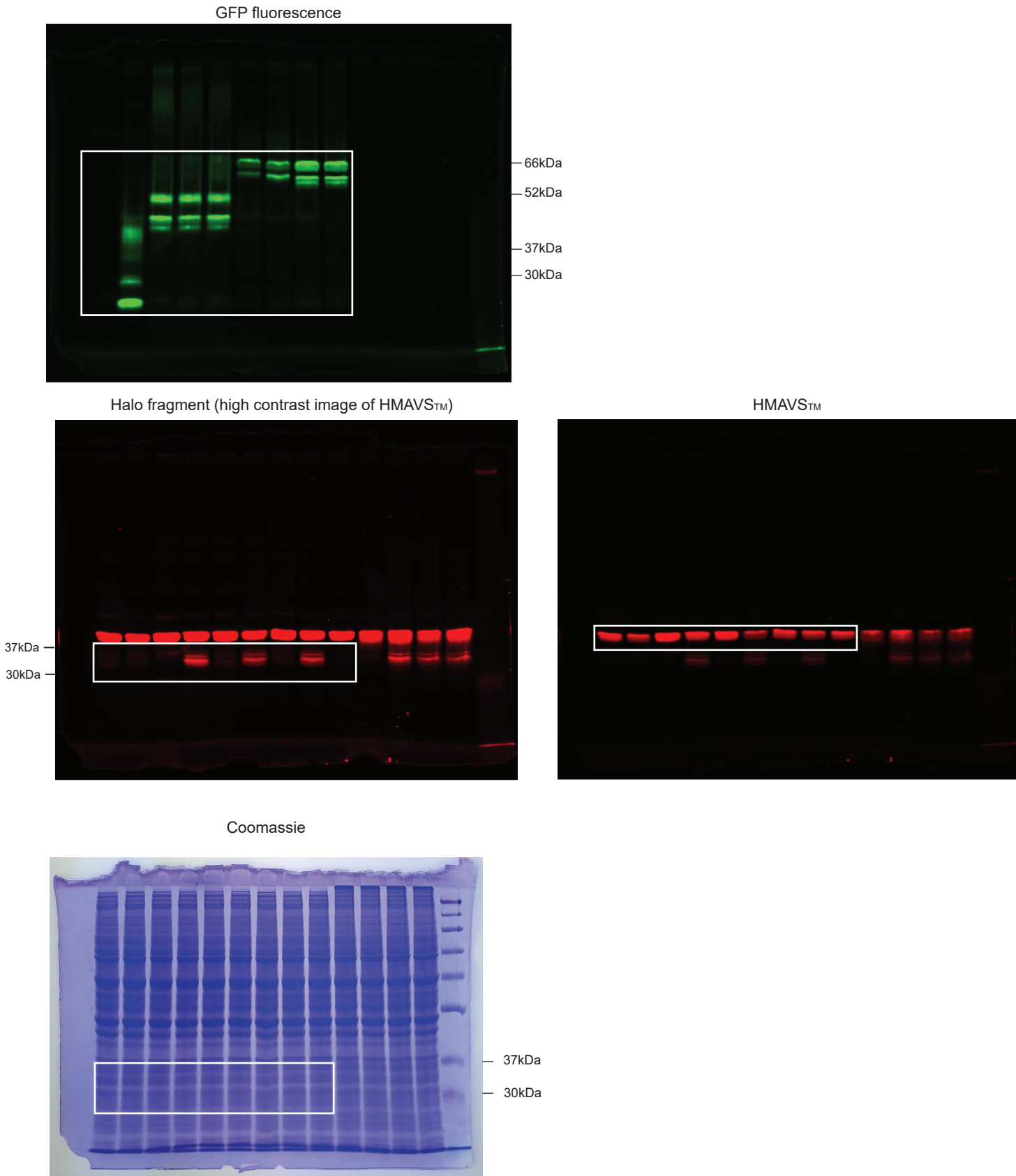

Supplement: Supplementary file 13 — Unprocessed western blots and/or gels. [file 41556_2025_1728_MOESM13_ESM.pdf]

Figure 7

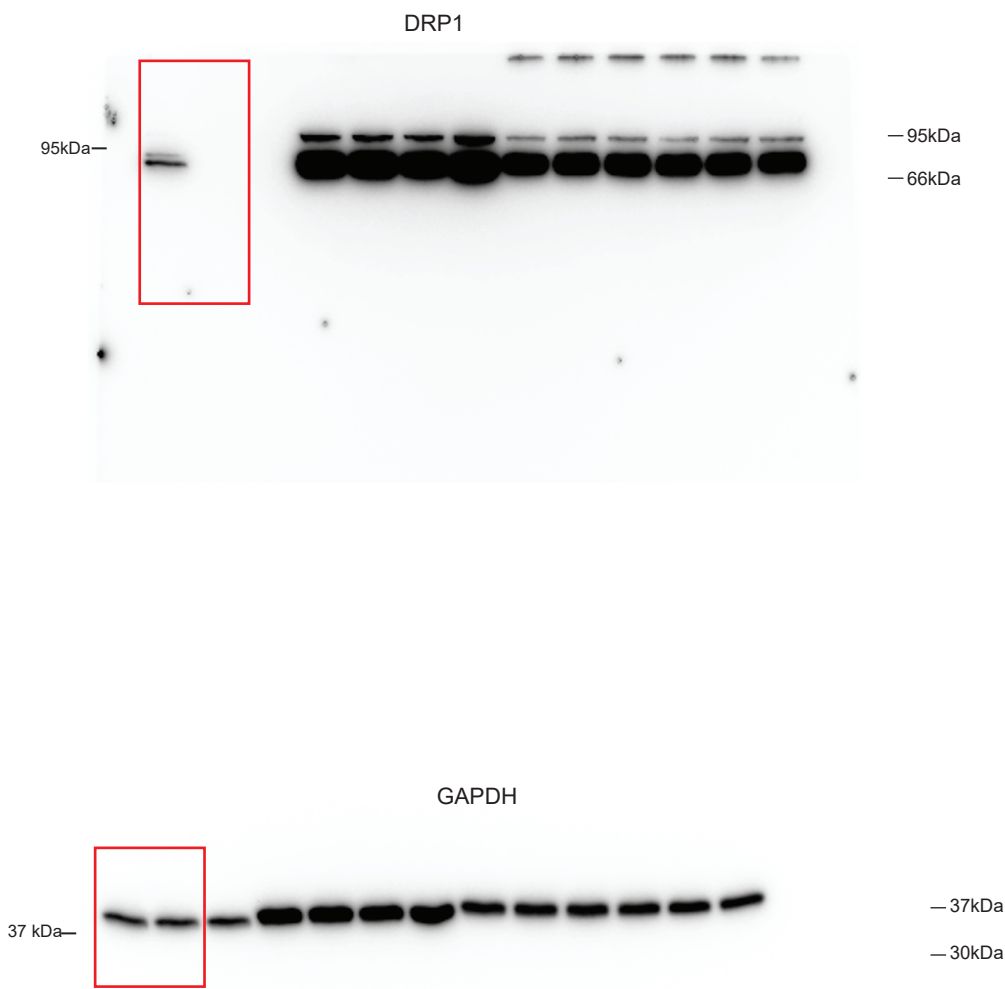

Supplement: Supplementary file 14 — Unprocessed western blots and/or gels. [file 41556_2025_1728_MOESM14_ESM.pdf]

Figure 8

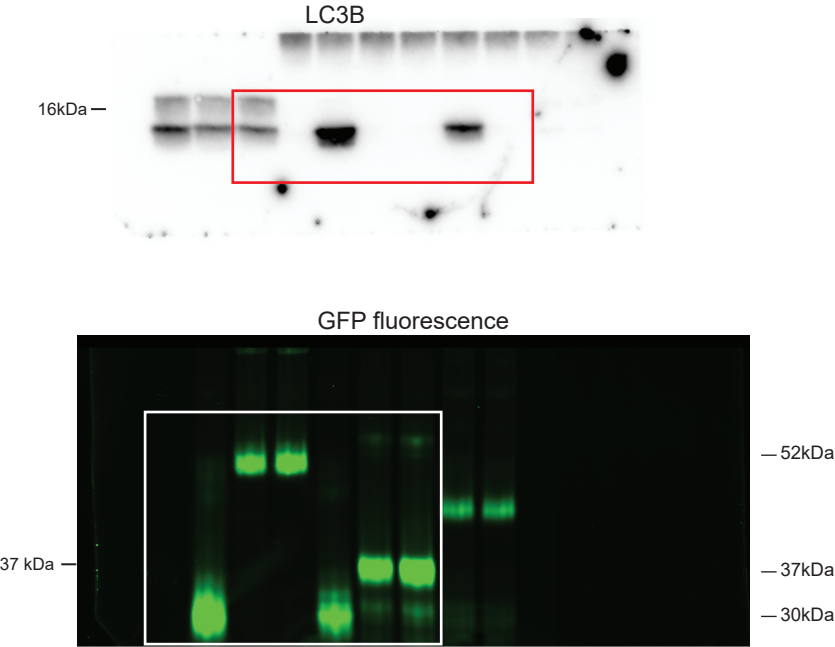

Supplement: Supplementary file 15 — Unprocessed western blots and/or gels. [file 41556_2025_1728_MOESM15_ESM.pdf]

Extended Figure 3

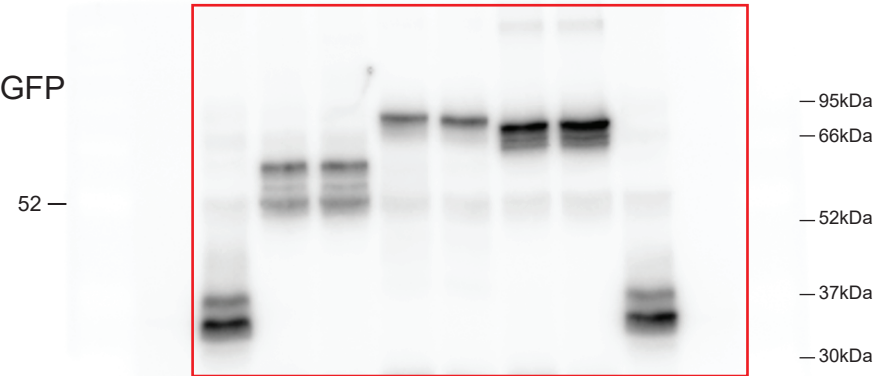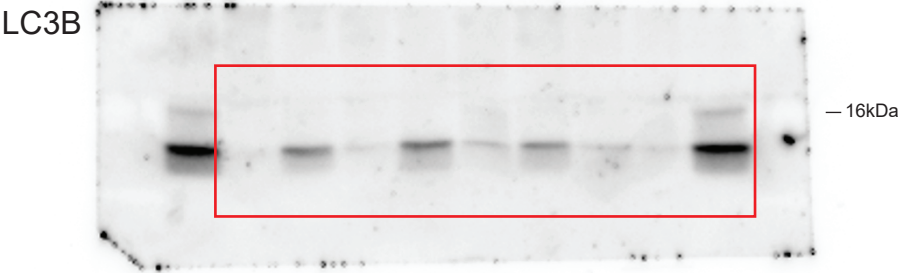

Supplement: Supplementary file 17 — Unprocessed western blots and/or gels. [file 41556_2025_1728_MOESM17_ESM.pdf]

Extended Figure 9

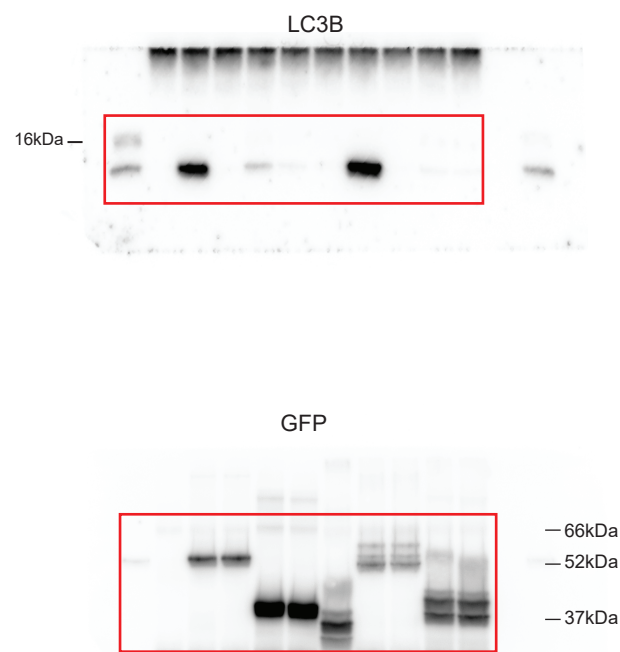

Supplement: Supplementary file 18 — Unprocessed western blots and/or gels. [file 41556_2025_1728_MOESM18_ESM.pdf]

Extended Figure 10

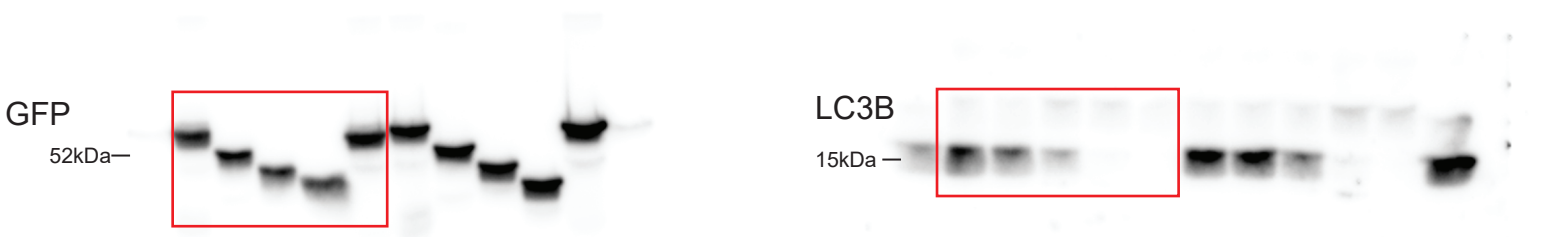

Supplement: Supplementary file 19 — Unprocessed western blots and/or gels. [file 41556_2025_1728_MOESM19_ESM.pdf]
